# Passion or Perseverance? The Effect of Perceived Autonomy Support and Grit on Academic Performance in College Students

**DOI:** 10.3390/ijerph17062143

**Published:** 2020-03-24

**Authors:** Elisa Huéscar Hernández, Juan Antonio Moreno-Murcia, Luís Cid, Diogo Monteiro, Filipe Rodrigues

**Affiliations:** 1 Psicología de la Salud Department, Edificio Altamira, Universidad Miguel Hernández de Elche, Avda. Universidad, s/n, 30202 Elche, Alicante, Spain; ehuescar@umh.es; 2 Sport Research Center, Universidad Miguel Hernández de Elche, Avda. de la Universidad, s/n, 03130 Elche, Alicante, Spain; 3 Sport Science School of Rio Maior (ESDRM—IPSantarém), Av. Dr. Mário Soares n-110, 2040-413 Rio Maior, Portugal; luiscid@esdrm.ipsantarem.pt (L.C.); diogomonteiro@esdrm.ipsantarem.pt (D.M.); frodrigues@esdrm.ipsantarem (F.R.); 4 Research Center in Sport Sciences, Health Sciences and Human Development (CIDESD), 5001-801 Vila Real, Portugal; 5 Life Quality Research Center (CIEQV), 2040-413 Santarém, Portugal

**Keywords:** teachers’ motivational styles, grit, self-determination theory, autonomy support

## Abstract

Background: Individuals who possess passion and perseverance to extensively work and study through challenges and adversity to achieve a set of goals are likely to reach higher achievement compared to others who lack similar facets. However, an under-researched question lingers over the effect of teacher-induced behaviors on academic outcomes such as grades and performance. The purpose of this study was to examine the relationships between teacher-induced autonomy support and student academic performance considering the mediating effect of basic psychological needs satisfaction, intrinsic motivation, and grit as two-independent factors. Methods: A convenience sample of 474 Sports Science students (*M_age_* = 21.83 years; *SD* = 3.91) participated in the study. All participants completed a multi-section survey assessing the variables under analysis. Results: The measurement and structural model displayed acceptable fit, hence direct and indirect effects were examined among the variables of interest. Basic psychological needs and intrinsic motivation seem to display a mediating role between perceived autonomy support and academic performance, through perseverance. Contrarily, grit-passion did not exhibit a significant indirect effect. Conclusions: Current results shed new insights on how perseverance can shape student motivation and school success considering the autonomy support induced by teachers.

## 1. Introduction

A relatively new term in the field of education, grit encompasses concepts of passion and perseverance and is becoming an increasingly important component in preparing students for academic success as well as for life. Grit has been associated with positive outcomes, in which teachers fostering grit enables students to work hard and stick to their achievement goals. Duckworth and Seligman [1,2] have shown that grit, a “symbiosis” between passion and perseverance, are better predictors of success in college compared to traditional SAT or IQ tests. In fact, there have been many studies attempting to show the importance of grit in achieving positive outcomes. However, a student belief’s system could be affected by how the need-supportive environment is perceived. Teachers could be a key role on determining the level of grit and, in turn, its predictability of academic aspects such as grades. 

The current study aims to fill this gap in the literature by taking different theoretical perspectives that could be more suitable for investigating the synergies of motivation and grit on student academic performance. As relatively little attention has been paid to how teacher-induced supportive behaviors interact with dispositional facets such as passion and perseverance, the goal of the present study was to examine the complex motivational sequence of motivation proposed by Ryan and Deci [3] on academic performance through the mediating role of grit. Moreover, we also investigated which grit factor displays the most significant predictability power on academic performance.

### 1.1. Conceptual Framework

Over the past two decades, considerable research has been directed towards identifying contributors to positive academic outcomes in students. One line of research has been grounded in self-determination theory (SDT), a macro theory of human motivation, concerned with understanding the natural tendencies to behave efficiently within an environment. This theoretical framework has been applied extensively in the educational domain [3], directing attention to teacher motivational styles and the effects of those styles on cognitive, behavioral and emotional outcomes. Considering this framework, Vallerand [4] established a motivational sequence, considering social factors as predictors of basic psychological needs (BPN) fulfillment, resulting in more self-determined motivation.

From this theoretical perspective, teachers should take into account the importance of supporting student autonomy [3]. Autonomy support is the interpersonal behavior teachers provide during class instruction to identify and build student sense of autonomy and inner motivational resources [5]. Hence, student perceived autonomy support from teachers refers to an atmosphere where students have volitional choice and are encouraged to express themselves in a positive manner [3]. According to past literature, BPN satisfaction are contingent on the experience of positive social factors; thus, higher levels of perceived autonomy-supportive behaviors would lead to higher levels of BPN satisfaction.

The BPN theory [3] is a mini-theory within the SDT framework, stating that the fulfillment of autonomy competence and relatedness will affect positively the tendency towards the integration of positive outcomes. The need for autonomy is considered to be the experience of will and psychological freedom. The need for competence refers to the need to interact effectively with the environment in order to experience capacities of producing desired outcomes and mastering a set of new skills. The need for relatedness implies to the need to feel connected with others having a sense of bellowing and positive relationships. The satisfaction of all three needs provide the basis for predicting positive outcomes, such as self-determined behavior [3]. This is an important aspect since the provision of need-supportive behaviors from teachers will determine the satisfaction of all BPN, leading to higher levels of student engagement towards classes. In fact, higher levels of BPN will ultimately predict the most self-determined regulation of motivation, intrinsic motivation in which the individual seeks out challenges and pleasure, and how the activity or behavior aligns with their sense of self [6].

### 1.2. Definition and Correlates of Grit

To date, a considerable amount of research conducted in the educational contexts has had the goal of understanding processes through which intrinsic motivation can be increased and strengthen academic performance [7]. However, what are the predictors of intrinsic motivation besides cognitive and behavioral outcomes? We highlight the fact that teacher adoption of an autonomy-supportive style in the classroom is not enough to understand academic achievement; it is likewise necessary to assess student dispositional facets towards their engagement and achievement in the classroom setting. Specifically, recent studies have examined grit as novel factor contributing to the explanation of academic performance. Within this line of research, there has been focused attention devoted to the qualities that contribute to long-term success [8] but less attention dedicated to understanding volitional student characteristics in any given situation [9].

According to Duckworth [10], grit is defined “as passion and perseverance for long-term goals”. Grit refers to the level of drive that individuals demonstrate in order to reach their goals over the long term and reflects trait-level perseverance and passion for long-term goals. Hence, grit involves working hard towards challenging objectives and maintaining effort and interest over years, despite failure, set-backs, and plateaus in progress. 

Grit is positively associated with adaptive outcomes among youth and adults, such as work satisfaction, career performance, and emotional outcomes [11]. Hence it could be also related to positive outcomes in the school-based context, promoting student achievement. In a recent study of adolescent students, Hagger and Hamilton [12] demonstrated the positive contribution of grit on student academic achievement. “Gritty” students were those who invested a great deal of time in remaining connected to task goals and thus were more likely to engage in deliberate behaviors that would enable them to achieve these goals. This line of research is consistent with expectations from grit theorists [13,14,15], showing the importance of measuring grit as a predictor of achievement. Of the two grit characteristics, research findings tend to indicate perseverance as a relatively stronger predictor in the attainment of long-term goals, including under adverse circumstances which would include objective failure or the feeling that one is not continuing to improve, compared to passion [10]. In line with these findings, Guo and colleagues [16] have found differentiated correlation patterns between grit factors and various psychological variables (e.g., motivation). Thus, we followed this assumption, considering passion and perseverance as two facets of grit, implying possible differentiated results in the classroom setting.

While some authors [17] consider assessing grit as an aggregated score to represent overall grit, others use separate scores for perseverance and passion in their analyses, in line with a recent meta-analysis [11]. In fact, the original grit scale was developed to measure two aspects of grit [18]. Thus, it is determined that there is a need for more studies that is specific to academic achievement considering both student dispositional traits.

### 1.3. Past Literature

Overall, the link between passion and perseverance and academic performance remains under-researched. Although grit would seem to be a fundamentally important quality in a dynamic context as what the educational setting is, the influence of other social forms that may interact with grit are not well understood. Some research indicate that student grit is influenced by teachers conduct during classes [10]. Specifically, how students perceived teacher-induced autonomy supportive behaviors. It is hypothesized that if a teacher finds the specific dispositional traits of each student and knows how to nurture the inherent interest of the students and reborn their proactivity towards learning activities, then a chain of positive outcomes can ensue. In accordance with the findings of Reeve and colleagues [19], it would be anticipated that teacher autonomy support for students would strengthen student intrinsic goals and reinforce student behaviors that is effortful, goal-directed, and reliant upon personal resources and intrinsic motivation [20]. 

Furthermore, research on the relative contributions of grit-perseverance and grit-passion to student academic performance outcomes have been limited. Existing literature has favorably generated the role of grit in contributing to adaptive academic outcomes [21], and literature has similarly been generated about the importance of teacher motivational behaviors in contributing to adaptive outcomes in students [22]. To our knowledge, however, investigations have yet to be conducted that considers both social factors and grit facets on student behavioral consequences, such as academic grades.

The bulk of research conducted to date has examined grit as only as a unitary variable and in consideration of its direct effect on student academic performance [23,24] and much remains to be known about the separate forms of grit [11]. A better understanding of the potential influence of grit between teacher need-supportive behavior and student academic achievement outcomes would help to clarify these effects [20].

#### Current Research

**Figure 1:**
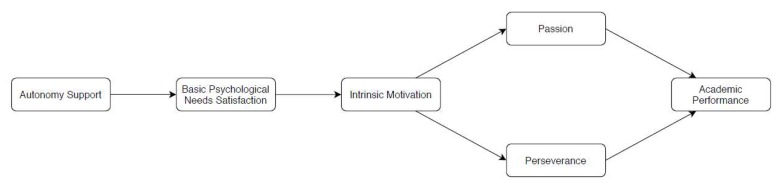
Hypothetical Model.

The purpose of the present study was to examine whether passion and perseverance in the classroom context plays a moderating role in the relationships between perceived autonomy supportive behavior and academic grades. Specifically, given the prominence of the SDT framework and grit on understanding achievement, a central aspect of the proposed model (see Figure 1) was to explore the potential value of the grit dimensions separately to understand how autonomy support is related to them, based on students experience of BPN satisfaction and intrinsic motivation. Based on previous research [1,5,13], we hypothesized that (i) perceived autonomy support would be positively associated with BPN satisfaction; (ii) the satisfaction of autonomy, competence, and relatedness as a unifying factor would predict positively intrinsic motivation; (iii) in turn, intrinsic motivation would predict positively both passion and perseverance, as two distinct forms of grit; (iv) grit factors would present a positive relationship with academic performance, assessed through grades; (v) grit-perseverance is anticipated to be display a more favorable effect on academic performance compared to grit-passion; and (vi), as a consequence, grit-perseverance would display a mediating role between perceived autonomy support and academic performance.

## 2. Materials and Methods 

### 2.1. Ethics Statement

Ethical approval for the research procedures was obtained from the lead author’s institutional body (Research Ethics Committee of Universidad Miguel Hernández de Elche, Spain, registration number DPS.JMM.01.17). Data collection procedures were conducted in accordance with the Declaration of Helsinki and its later amendments. Informed consent was obtained individually by each participant.

### 2.2. Participants

A non-probabilistic convenience sample of 443 Sports Science students (female = 135; male = 359) from eight different Portuguese universities aged between 18 and 28 years (*M* = 22.47, *SD* = 2.78) were recruited. For eligibility, potential participants needed to be aged above or equal 18 and below or equal 30 years and be registered in any class on the year of data collection.

### 2.3. Measures

All instruments previously not validated in Portuguese were translated to Portuguese and back-translated to the original scale based on the protocol outlined by several authors [25,26]. We examined the factor structure of each scale before testing the hypothesized model.

#### 2.3.1. Autonomy Support 

The Autonomy Support Scale [27] was used to measure teacher-induced autonomy support students perceived during class. This 12-item (one factor) scale assesses autonomy-supportive behaviors (e.g., “*Throughout the class the instructor invites us to make suggestions and values our ideas and suggestions*”), which are preceded by the sentence “*My teacher…*”. Participants responded to each item using a 5-point scale anchored from 1 (totally disagree) to 5 (totally agree). The psychometric proprieties of the scale for this study provided an acceptable fit to the data (CFI = 0.924; TLI = 0.905; SRMR = 0.073, RMSEA = 0.061 (CI 90% = 0.067, 0.079)).

#### 2.3.2. Basic Psychological Needs

The Basic Psychological Need in Exercise Scale Portuguese version [28] was employed to measure the extent to which participants felt that their needs were met during classes. This 15-item (5 items each factor) examines autonomy (“*I feel that I can complete task in the way that I prefer*”), competence (“*I believe that I can complete personal challenges*”) and relatedness (“*I feel that I get along well with others when I engage in the activities with others*”) satisfaction. Participants responded to each stem on a 6-point scale anchored with endpoints of 1 (true) and 5 (false). This scale has shown to be a reliable source of BPN measurement [29]. The psychometric proprieties of the scale for this study provided an acceptable fit to the data (CFI = 0.985; TLI = 0.975; SRMR = 0.034, RMSEA = 0.041 (CI 90% = 0.037, 0.050)).

#### 2.3.3. Intrinsic Motivation 

The Academic Motivation Scale [30] was used to measure how students perceive their intrinsic motivation towards learning. Specifically, four items measuring intrinsic motivation (e.g., “*For the pleasure I feel in expanding my knowledge about topics that interest me*”) were considered for this study. Items were preceded by the stem “*Why do you study?*” and participants indicated their level of agreement with each item using a 7-point scale ranging from 1 (totally disagree) to 7 (totally agree). The psychometric proprieties of the scale for this study provided an acceptable fit to the data (CFI = 0.946; TLI = 0.930; SRMR = 0.041, RMSEA = 0.042 (CI 90% = 0.039, 0.049)).

#### 2.3.4. Passion and Perseverance 

The Grit Scale short version was used to measure passion and perseverance students have towards the classroom setting [31]. Items were adapted to the classroom context, namely grit for passion (“*I often set a goal but later choose to pursue a different one*”) and grit for perseverance (“*Setbacks do not discourage me. I do not give up easily*”). Participants responded to eight items (four each factor) using a 5-point scale anchored from 1 (totally disagree) to 5 (totally agree). The psychometric proprieties of the scale provided an adequate fit to the data (CFI = 0.947; TLI = 0.923; SRMR = 0.053, RMSEA = 0.041 (CI 90% = 0.027, 0.039)).

#### 2.3.5. Academic Performance

Weighted grade point average was obtained for each student at the end of the first academic year from the school records. This measure ranges from 0 to 20, zero (0) being the lowest score and twenty (20) the highest score possible during an academic year according to the Portuguese School System. The validity and reliability of this measure has been evidenced in previous research [32] and is considered to be a robust school performance indicator [3].

### 2.4. Procedure

Data was collected during the school year by the researchers who were available to answer any questions potential participants could have. School boards were personally contacted, objectives were explained, and permission was obtained to conduct this study. Teachers were contacted afterwards, and the objectives were explained. Before class, researchers explained the purpose of this study to each student and confidentiality was explained and ensured. Those students who participated voluntarily provided informed consent. Students completed an anonymous multi-section questionnaire which was accessible to them through Google Forms, which took nearly 15 minutes to complete in quiet classroom conditions.

### 2.5. Data Analysis

Descriptive statistics (means, standard deviations, skewness, and kurtosis values) and correlations among variables were calculated. In accordance with Kline’s [33] recommendations, a two-step model assessment was performed using Mplus 7.4 [34]. The Maximum Likelihood Robust (MLR) estimator was used since it is robust to non-normality and non-independence of observations, while also being sensitive to missing data considerations [35].

A Confirmatory Factor Analysis (CFA) was performed at the first step to test measurement model fit. Then the Average Variance Extracted (AVE) scores were calculated as an estimate of convergent validity with values ≥ 0.50 considered to be acceptable [36]. Discriminant validity was confirmed when the AVE scores were higher than the squared correlations (r*^2^*) values across constructs [36,37]. Composite Reliability (CR) coefficients were calculated using the Raykov formula [37] considering scores ≥0.70 as acceptable.

A structural equation model (SEM) was then tested on the hypothesized model to assess direct and indirect effects among the factors. Chi-square (χ^2^) test statistics are commonly used for examining model fit. However, due to its sensitivity to sample size and model specification [29], we considered the following traditional and incremental indices for assessing model adequacy: the Comparative Fit Index (CFI), Tucker-Lewis Index (TLI), Standard Root Mean Residual (SRMR), and Root Mean Square Error of Approximation (RMSEA) with its respective confidence interval (CI 90%). For these indices, values on CFI and TLI ≥ 0.90 and values for the SRMR and an RMSEA ≤ 0.80 were considered acceptable [36,37,38]. Direct and indirect effect were considered to be significant (*p* ≤ 0.05) if its 95% confidence interval (CI 95%) did not include “0” [39,40]. 

## 3. Results

### 3.1. Preliminary Analysis

Table 1 presents the descriptive statistics, composite reliability coefficients, AVE scores, and bivariate correlations for all the variables under analysis. In this study, the mean scores for all variables were above the scale mid-point. Perseverance displayed a higher mean score compared to passion. Univariate skewness and kurtosis were contained within cutoffs (−2.0 and +2.0, and −7 and +7, respectively). All factors displayed an acceptable internal consistency since the CR coefficients were above acceptable. According to the correlation matrix, autonomy support was positively correlated with BPN satisfaction, intrinsic motivation, and perseverance, but not with passion and academic performance. Similar correlations were found between BPN satisfaction, intrinsic motivation, and perseverance. Both grit factors displayed a significant positive correlation with academic performance, perseverance being the factor with the highest correlation coefficient (0.20). AVE scores were above 0.50 achieving convergent validity, and discriminant validity was also confirmed since the r^2^ for each correlation was below AVE. For more details see Table 1.

### 3.2. Measurement and Structural Model Fit

**Figure 2:**
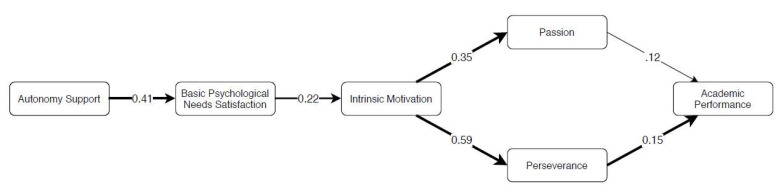
Standardized coefficients are presented; thick lines = significant at *p* ≤ 0.05.

The measurement model did provide adequate fit to the data: (χ2 = 451.308 (310), *p* ≥ 0.001; CFI = 0.959, TLI = 0.953, SRMR = 0.043, RMSEA = 0.031 (CI 90% = 0.025, 0.037)). Thus, we moved forward on testing the structural model. The SEM model (Figure 2) also indicated acceptable fit to the data: (χ^2^ = 546.378 (319), *p* ≥ 0.001; CFI = 0.93, TLI = 0.927, SRMR = 0.080, RMSEA = 0.039 (CI 90% = 0.033, 0.044)). 

As theoretically proposed, positive and significant direct effects were found among variables, namely, autonomy support predicted BPN satisfaction; BPN satisfaction predicted intrinsic motivation; and intrinsic motivation, in turn, predicted both grit factors (i.e., passion and perseverance). Grit-perseverance, but not grit-passion, was significantly and positively related to academic performance. Looking at the indirect effects, all paths were positive and significant (Table 2). Hence, we move forward on examining specific indirect paths, considering autonomy support as the independent variable predicting academic performance.

Accounting for indirect paths, all were significant via grit-perseverance, but not via grit-passion (Table 3). These findings reinforce previous direct effect analysis, showing that grit-passion seems to now stand as a significant predictor of academic performance nor does it seem to play a mediating role between autonomy support, BPN satisfaction, and intrinsic motivation on academic performance. For detailed information see Table 3.

## 4. Discussion

The present study provided several important findings that further our knowledge of the effect of grit on academic performance in sport science students. Specifically, perseverance, and not passion, displayed a mediating role between perceived autonomy-supportive behaviors and grades. Consistent with our initial hypothesis, current results supported expectations from current motivational perspectives [5,13]. We discuss the particularities of these findings, as well as the study limitations, in the following paragraphs.

First of all, the findings indicated the presence of a positive and significant relationship between teacher-induced autonomy support and the satisfaction of basic psychological needs in students and each was significantly related to intrinsic motivation. Specifically, current results revealed college students to highly perceive autonomy-supportive behaviors predicting higher levels of BPN satisfaction. These results indicated that the social environment created by the teacher in the classroom context is crucial to promote needs fulfillment. Taken together, teacher actions that originate student self-volitional choice, giving explanatory rationales and enhancing student internal perceived locus of causality, is essential to satisfy all three needs based on the theoretical framework of SDT [5]. 

The relationship between BPN satisfaction and self-determined motivation is well documented in the literature [3,5,41]. Our findings further inform the existing prediction of autonomy, competence, and relatedness satisfaction on intrinsic motivation. With respect to intrinsic motivation, initial works examining the relationship between student grit and their academic motivation have revealed that individual differences in grit can have consequences in the realization of adaptive learning outcomes when students perceive that they are receiving teacher support [20]. Hence, self-determined students are more likely to feel more autonomously motivated to achieve and endure during hard work. In other words, students who intentionally engage and put effort on their task know that it will bring them closer towards their achievement goals. This relates to current results, showing a higher prediction of intrinsic motivation on perseverance compared to passion. Being able to experience pleasure, personal interest, and express intrinsic motives tend to lead students to higher levels of resilience and self-directed rewards.

In recent years, grit has been identified as an individual variable that can contribute to academic success and favorable psychosocial outcomes. There also has been progress in the area of measuring grit as a two-dimensional factor measure [11]. This trend reflects the increased use of multidimensional models to explain academic achievement. Related to this, the perseverance dimension of grit has been recognized as the grit dimension most commonly associated with these outcomes [21]. Current results provided support for our hypotheses. Specifically, the findings from our study suggested that the two dimensions of grit were not of equal importance, grit-perseverance being a significant predictor of academic performance but grit-passion not.

Perseverance seems to predict academic success, showing that students who are encouraged to value their hard work are more prone to display higher academic grades. In other words, teachers promoting self-determined drive and perseverance could help students succeed in school and maintain higher grades throughout their academic courses. Although speculative, the literature tends to support these associations [42]. Students should perceive teachers as need-supportive figures, to endorse in self-regulated behaviors, in turn leading to adaptive outcomes.

To date, we are not aware of other research that has examined the relationships between perceived autonomy support along with grit facets and academic success. Even though research on autonomy support and grit is quite limited [43], these results are consistent with the studies that have addressed the role of intrinsic motivation in the educational process [41] and highlight the importance of student persistence and effort in achievement contexts [44]. A study conducted by Scharneck [43] recently found that student-athletes’ perception of autonomy support was positively related to grit through the mediating influence of intrinsic motivation. This line of investigation indicates that social influences can be powerful in shaping feelings of autonomy.

These results shine new light on the relative influence of the grit dimensions on student academic achievement and highlights the influence of teacher motivational styles in contributing to these outcomes. Of the grit dimensions, grit-perseverance was found to be more essential to academic achievement than grit-passion. Although there has been some research that has examined the unique influence of grit and autonomy support on student achievement [45,46], more research is necessary to better understand the interactive effects of student grit characteristics and teacher need-supportive behaviors on student academic performance. It is hypothesized that when students feel more self-determined in the learning process, they simultaneously demonstrate a stronger desire to complete quality work, putting in effort and hard work. 

Much remains to be known on this topic, but it seems that when teachers are perceived as autonomy-supportive figures, student are influenced in a way that facilitates their academic outcomes. The nature of such a learning context would thus be congruent with the objectives of those students who desire to rely upon their personal effort to succeed and who have a strong personal interest or connection to the domain of interest [47].

### Limitations

Despite the analyses, methods, and novel approach to understanding the mediating role of grit and its relationships with motivational tenets and academic performance, the current study still has limitations that should be addressed. First, the cross-sectional nature of the collected data. Longitudinal designs would be beneficial to examine how these relationships play out over time. As previously reported, longitudinal designs could share new insights on how teacher-induced need-supportive behaviors vary over time [48] and how this impacts student grit towards academic performance. Second, this study was conducted with a sample of Portuguese college students. Therefore, our findings cannot be generalized to other countries or other contexts, as more research is needed to establish an invariant multidimensional model. Third, only a “bright” side of motivation was considered. Specifically, we only attended to autonomy-supportive behaviors, BPN satisfaction, and intrinsic motivation. Thus, future studies should assess the possibility of specific paths between need-supportive and need-thwarting behaviors on BPN satisfaction and also needs frustration on cognitive and behavioral outcomes. Recent studies [49] have shown that motivational sequences co-occur, displaying significant differences among a bright and a dark side of motivation. Given the ongoing importance of understanding the predictability of grit in cognitive and behavioral outcomes, this study opens doors to new research questions on how grit experimentally manipulated could account for changes in college grades. Last, grit has been related to the social-emotional learning of individuals. The development of social emotions does not rule out the possibility that the background of each individual, such as culture and economic status, could affect their performance in school. Although most of our sample was Caucasian, other socio-demographic factors (e.g., economic status, cultural background) should be considered in future research.

## 5. Conclusions

This study has provided evidence from the motivational and grit mechanisms that underpin academic performance. In sum, the current study makes a unique contribution to the literature by assessing the mediating effect of grit, specifically perseverance, in the relationship between autonomy support and student academic performance. The study found evidence that teachers are important influencers on the environmental cues about BPN satisfaction, intrinsic motivation, dispositional factors (i.e., grit), and academic performance. The importance of the need-supportive environment to student motivation is well-documented in the literature [50,51] but the current study shows that more research is needed since the literature would benefit from examining in more detail grit as independent factors, if we are to create favorable climates that optimize student performance and wider academic benefits for young sport science college students. All in all, these findings have important implications for instructional practice and highlight the importance of increasing teaching skills to provide autonomy support to students, particularly for college students with low resilience and perseverance levels [47,52].

The current study manifests the need for teachers to become more self-aware of the importance of self-regulated learning and can be trained through the implementation of strategies that focus on promoting a sense of grit towards student engagement in the classroom setting [53]. Teachers should look at failure as a data point rather than a normative indicator of student motivation. Therefore, special attention should be paid to the teacher’s role as a decision maker in the classroom setting. These actions could include enhancements of collaborations among students, less focus on inter-competitive culture, providing constructive comments and feedback, and also improvement of creativity and innovations. Teachers also should understand the necessity for perseverance, designing learning experiences that challenge college students (in this study, sport science individuals). Hence, teaching for grit means taking the long-term perspective, looking to develop and increase student academic performance, but also the outer-school perspective. Understanding the crucial role of grit and how we can foster it increases the likelihood that students (as well as teachers) will succeed.

## Figures and Tables

**Table 1:** Descriptive statistics, Composite Reliability, Average Variance Extracted, and correlations.

	M	SD	S	K	CR	AVE	Correlations				
							1.	2.	3.	4.	5.
1. Autonomy Support	4.18	0.55	−1.50	4.8	0.89	0.65					
2. BPN Satisfaction	4.01	0.50	−0.56	1.76	0.79	0.77	0.39 ** (0.15)				
3. Intrinsic Motivation	5.38	1.12	−0.69	0.41	0.84	0.75	0.46 ** (0.21)	0.17 ** (0.03)			
4. Grit-Passion	2.98	0.83	0.34	−0.17	0.70	0.61	0.10 (0.01)	−0.02 (0.00)	0.33 ** (0.11)		
5. Grit-Perseverance	4.05	0.57	−0.53	0.38	0.71	0.61	0.33 ** (0.11)	0.22 ** (0.05)	0.57 ** (0.32)	0.46 ** (0.21)	
6. Academic Performance	13.16	1.31	0.12	0.31	-	-	−0.01 (0.00)	−0.02 (0.00)	0.18 ** (0.03)	0.15 ** (0.03)	0.35 ** (0.04)

Note: M = Mean, SD = Standard Deviation, S = Skewness; K = Kurtosis, CR = Composite Reliability; AVE = Average Variance Extracted; squared correlation in brackets; ** *p* = 0.01.

**Table 2:** Path estimates.

Path	β	CI 95%	
		Lower	Upper
**Direct effect**			
Autonomy Support → BPN Satisfaction	0.45	0.26	0.59
BPN Satisfaction → Intrinsic Motivation	0.26	0.06	0.38
Intrinsic Motivation → Passion	0.28	0.19	0.52
Intrinsic Motivation → Perseverance	0.64	0.45	0.73
Passion → Academic Performance	0.10	−0.05	0.30
Perseverance → Academic Performance	0.20	0.05	0.26
**Indirect effect**			
Autonomy Support → Academic Performance	0.01	0.00	0.02
Autonomy Support → Passion	0.03	0.01	0.05
Autonomy Support → Perseverance	0.05	0.02	0.09
Autonomy Support → Intrinsic Motivation	0.09	0.04	0.14
BPN Satisfaction → Academic Performance	0.03	0.01	0.05
BPN Satisfaction → Passion	0.08	0.04	0.12
BPN Satisfaction → Perseverance	0.13	0.06	0.20
Intrinsic Motivation → Academic Performance	0.14	0.08	0.19

Note: β = standardized coefficient; CI 95% = Confidence Interval at 95%.

**Table 3:** Specific indirect paths.

Path	β	CI 95%	
		Lower	Upper
AS → BPNS → IM → PAS → AP	0.00	−0.01	0.02
AS → BPNS → IM → PER → AP	0.09	0.00	0.11
BPNS → IM → PAS → AP	0.00	−0.01	0.02
BPNS → IM → PER → AP	0.06	0.01	0.10
IM → PAS → AP	0.04	−0.01	0.08
IM → PER → AP	0.12	0.03	0.18

Note: AS = Autonomy Support; BPNS = Basic Psychological Need Satisfaction; IM = Intrinsic Motivation; PAS = Passion; PER = Perseverance; AP = Academic Performance; β = standardized coefficient; CI 95% = Confidence Interval at 95%.
